# Mortality Trend of Hematological Neoplasms in Babol, Northern Iran (2013-2021)

**DOI:** 10.34172/aim.31147

**Published:** 2024-12-01

**Authors:** Pouyan Ebrahimi, Mohammad-Amin Ghezel, Seyed-Hossein Hosseini-Berneti, Amir-Hossein Lashkarbolouki, Mohsen Karami, Hossein-Ali Nikbakht

**Affiliations:** ^1^Student Research Committee, Babol University of Medical Sciences, Babol, Iran; ^2^Infectious Diseases and Tropical Medicine Research Center, Health Research Institute, Department of Parasitology and Mycology, Babol University of Medical Sciences, Babol, Iran; ^3^Social Determinants of Health Research Center, Health Research Institute, Babol University of Medical Sciences, Babol, Iran

**Keywords:** Hematologic neoplasms, Iran, Leukemia, Lymphoma, Mortality

## Abstract

**Background:**

Blood cancers account for a significant proportion of cancer-related deaths worldwide. In this study, hematological cancer mortality in northern Iran was examined during 2013-2021, along with age-adjusted mortality rates.

**Methods:**

In a cross-sectional study, we conducted an analysis of all deaths from hematological neoplasms registered in Babol city between 2013 and 2021. In order to estimate the population, the most recent census data was used. The mortality rates and trends for each hematological malignancy were reported in crude mortality rate (CMR) and age-standardized mortality rate (ASMR).

**Results:**

In total, 357 deaths (10.8% of all cancer-related deaths) were attributed to hematological neoplasms, with an average age of 61.9±19.3 years. The crude and age-adjusted mortality rates of hematological neoplasms increased from 3.1 and 2.7 per 100000 people in 2013 to 8.1 and 6.9 per 100,000 people in 2021, respectively. Mortality trends of hematological cancers increased with age decade for both sexes (*P*<0.001). Additionally, when examining the trends of each hematological neoplasm, there was a significant increase in neoplasms including non-Hodgkin lymphoma (*P*=0.033), multiple myeloma (*P*=0.002), and leukemia (*P*<0.001), except for the consistent trend observed in Hodgkin lymphoma (*P*=0.247).

**Conclusion:**

The trend of hematological malignancies in Babol city is increasing across all age groups and in both sexes. This study emphasizes the need for effective prevention and treatment strategies, including improving access to cancer care, enhancing surveillance in families with blood malignancies and reducing modifiable risk factors. Additionally, further research is warranted to develop targeted interventions.

## Introduction

 Tumor formation occurs due to excessive cellular growth and proliferation. Hematological cancers represent a significant cause of mortality globally.^1^ There were an estimated 1.3 million new cases of hematological malignancies worldwide in 2020, resulting in 700 000 deaths.^2^ The incidence of most hematological cancers rises with advancing age.^3^ According to the Global Burden of Disease (GBD) study, the incidence of non-Hodgkin’s lymphoma (NHL) and leukemias elevated by 45% and 26% from 2006 to 2016, respectively.^4^

 Hematological cancers encompass a variety of blood diseases that impact blood cells and tissues involved in blood formation.^5^ This group of cancers includes leukemia, lymphoma, and myeloma, each characterized by distinct features, pathogenesis, and treatment outcomes. In recent decades, there has been extensive research on the molecular and genetic mechanisms influencing the development and progression of hematological cancers.^6^ This research has resulted in identifying novel targeted therapies and immunotherapies capable of selectively targeting pathways in cancer cells while sparing healthy cells.^7,8^ Significant recent advances have been made in the treatment of certain blood cancers such as Hodgkin’s lymphoma (HL) and non-Hodgkin’s lymphoma (NHL), chronic myeloid and lymphocytic leukemias (CML and CLL), and plasma cell myeloma (PCM).^5,9-11^

 Analyzing the causes of death in individuals with leukemia is a complex endeavor. As an example, the number of deaths due to certain cancers like HL has declined, but the number of deaths due to new cancers, like AML, and treatment-related cardiovascular diseases has increased.^12,13^ Cancer survivors are susceptible to a broad spectrum of late treatment-related side effects, which can significantly impact their prognosis. In older individuals with hematological cancers, improved survival following cancer diagnosis may contribute to elevated mortality rates from age-related conditions such as cardiovascular disease and diabetes mellitus.^14^ Despite substantial progress in cancer treatment and diagnosis, significant challenges persist in enhancing patient survival rates and mitigating treatment-related side effects.^15^

 In every country, it is imperative to design economic, social, and health programs that aim to increase life expectancy and reduce mortality rates.^16,17^ Analyzing mortality data also evaluates the effectiveness and performance of cancer preventive and treatment strategies. In Iran, the Civil Registration and Vital Statistics System (CRVS) is currently implemented in 30 provinces in order to record and classify deaths based on their causes. It is the best data source for estimating cancer mortality.^16^

 With a population of over 500 000, Babol is the second-most populated city in the Mazandaran province. As hematological disorders are an important part of public health and as cancer incidence and mortality trends change, the purpose of this study is to compare hematological cancer mortality trends in Babol with regional and global statistics over a nine-year period (2013-2021).

## Materials and Methods

 In this cross-sectional study, all deaths registered and classified as hematological neoplasms by the Babol University of Medical Sciences deputy health department between 2013 and 2021 were analyzed. The research protocol with code (MUBABOL.HRI.REC.1401.153) has been approved by the ethics committee of Babol University of Medical Sciences.

 Mortality data were collected from the department responsible for registering and classifying causes of death, which receives information from reliable sources such as mortuaries, forensic medicine, hospitals, and physicians trained in death registration. All cases undergo review by relevant experts in the health department and are entered into the Ministry of Health’s online death registration system. Following consultation with the Health Dean of Babol University of Medical Sciences, permission was obtained to collect data on hematological neoplasm-related deaths.

 Then, these data were reviewed for duplicate entries, controlled against other registered information, improbable cause-of-death codes based on gender and age, and were examined for whether the cause was known, and codes for poorly defined or empty cause-of-death categories, and corrected if necessary. Additionally, cause-of-death data were revised by relevant experts through accessing relevant records. After duplicate entries were removed, and poorly defined or empty codes were redistributed, the data were validated by program supervisors at the Health Ministry, enabling final referencing and reporting.

 The Tenth Revision of the International Classification of Diseases and Related Health Problems (ICD-10) was used to categorize the cause of death.^18^ Additionally, the coding classification was based on the GLOBOCAN 2020 classification^19^ using the same coding system. Therefore, the cancer codes studied are as follows: Hodgkin lymphoma (HL) (C81), non-Hodgkin lymphoma (NHL) (C82-86, C96), multiple myeloma (C88 + C90), and leukemia (C91-95).

 Data analysis was performed using the SPSS (version 22) and STATA (version 14) software. Data were presented as means and standard deviations for quantitative data; however, frequency and percentages were used for qualitative data. Census data from the Statistical Center of Iran were used to calculate crude mortality rates (CMRs) by age groups. Additionally, the standard population presented in the GLOBOCAN database provided by International Agency for Research on Cancer (IARC) was used to calculate age-standardized mortality rates (ASMR) per 100 000 individuals using direct standardization. As a result, the crude and age-standardized rates (ASRs) were reported along with 95% confidence intervals (CIs). An analysis of the mortality trend over the study period was conducted using the Cochran-Armitage-Trend Test, considering a significance level of P < 0.05.

## Results

 Between 2013 and 2021, a total of 3,294 cancer deaths were recorded in Babol city. Of these, 357 deaths (10.8%) were attributed to hematological neoplasms. Among these, 233 (65.3%) were male, 171 (47.9%) were urban residents, and 83 patients (23.2%) were under 50 years of age. NHL was the most common hematological neoplasm with 140 deaths (39.2%), followed by leukemia with 127 deaths (35.6%), HL with 46 deaths (12.9%), and multiple myeloma with 44 deaths (12.3%), respectively.

**Figure 1:**
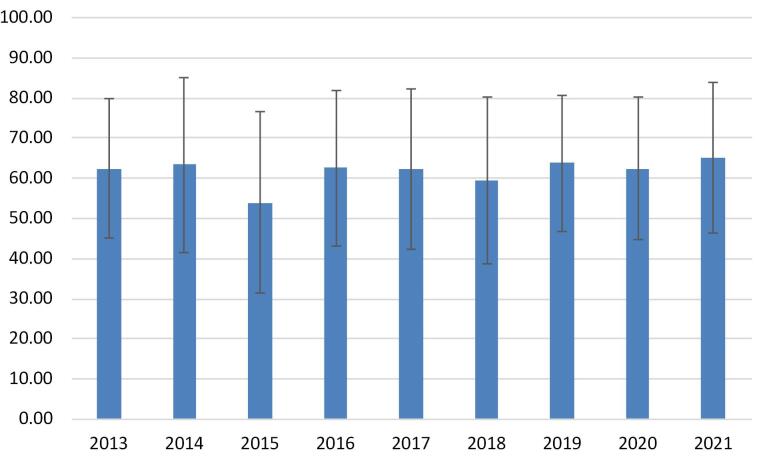
Mean (Standard Deviation) Age of Hematological Neoplasms between 2013-2021 in Babol City

 The mean age of patients who died due to hematological neoplasms was 61.9 ± 19.3 years (range: 2-99 years), with 83 patients (23.2%) under 50 years of age. The lowest and highest mean age of patients pertained to 2015 (53.9 ± 22.5 years) and 2021 (65.0 ± 18.7 years) (Figure 1).

**Figure 2:**
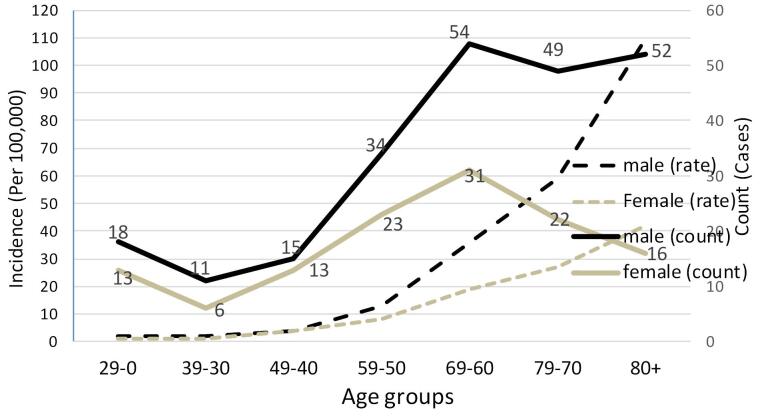
Examination of the Trend in Mortality Rate and Incidence Per 100,000 Population Due to Hematological Neoplasms by Age Categories in Babol (Iran) During 2013-2021 (*P* value trend < 0.001 for males and females)

 Based on age group analyses, the mortality rate for hematological neoplasms increased consistently in both genders (*P* < 0.05) (Figure 2).

**Figure 3:**
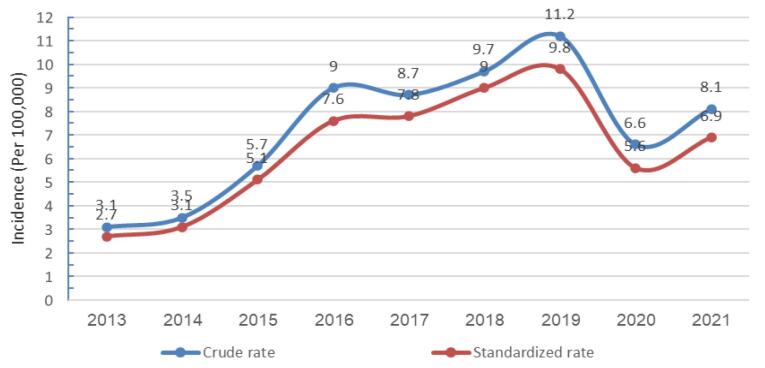
Trend of Crude and Age-Standardized Mortality Rates of Hematological Neoplasms per 100 000 Population in Babol (Iran) 2013-2021

 The CMRs in all cases were higher compared to the ASMR for hematological neoplasms. The highest CMR (11.2 per 100 000 people) and ASRs (9.8 per 100 000 people) were observed in 2019. Additionally, the lowest CMR (3.1 per 100 000 people) and ASRs (2.7 per 100 000 people) were recorded in 2013. Hematological neoplasms had CMR and ASR of 3.1 and 2.7, respectively, in 2013. By 2021, these values had risen to 8.1 and 6.9 per 100 000 people, showing a statistically significant increase (*P* < 0.001) (Figure 3).

 In examining the mortality rate of hematological neoplasms by gender and year, 2019 had the highest CMR and ASRs in men, with 14.3 and 12.9 per 100 000 people, respectively, and 8.0 and 6.7 per 100 000 people in women, respectively. The lowest mortality rates for both men and women, with crude and age-adjusted rates of less than 4.0 per 100 000 people, were recorded in the years 2013 and 2014, respectively.

 In analyzing the trend of mortality by gender, both CMR and ASRs increased significantly in men from 3.9 and 3.6 per 100 000 people in 2013 to 10.5 and 8.4 per 100 000 people in 2021, respectively (*P* = 0.001). A significant increase was also observed in the mortality trend among women, for whom the CMR and ASRs increased from 2.4 and 1.9 per 100 000 people in 2013 to 5.7 and 5.2 per 100 000 people in 2021, respectively (*P* = 0.003) (Table 1).

**Table 1:** Study Years and Gender-based Mortality Rates of Hematological Neoplasms in Babol (Iran) 2013-2021

**Years**	**Male**			**Female**		
	**CMR**	**ASMR**		**CMR**	**ASMR**	
		**Rate**	**95% CI**		**Rate**	**95% CI**
2013	3.9	3.6	1.3 – 5.9	2.4	1.9	0.4 – 3.4
2014	5.0	4.3	1.9 – 6.7	1.9	1.9	0.2 – 3.5
2015	8.7	7.4	4.2 – 10.6	2.7	2.7	0.7 – 4.8
2016	10.5	9.0	5.6 – 12.5	7.6	6.3	3.5 – 9.2
2017	11.8	10.1	6.5 – 13.8	5.6	5.4	2.6 – 8.1
2018	12.7	11.4	7.5 – 15.3	6.6	6.5	3.4 – 9.6
2019	14.3	12.9	8.8 – 17.1	8.0	6.7	3.8 – 9.5
2020	7.8	6.8	3.8 – 9.8	5.4	4.4	2.1 – 6.7
2021	10.5	8.4	5.3 – 11.6	5.7	5.2	2.6 – 7.8

CMR: Crude mortality rate; ASMR: Age-standardized mortality rate; CI: Confidence interval. ^*^P-value trend for female = 0.003 and male = 0.001

 In the examination of the trend of mortality for hematological neoplasms, NHL showed a significant increase from 0.2 per 100 000 people in 2013 to 1.9 and 1.7 per 100 000 people, respectively (*P* = 0.033). Additionally, a significant increasing trend was observed in multiple myeloma, with CMR and ASRs of 0.2 per 100 000 people in 2013 and 1.2 and 1.1 per 100 000 people in 2021, and leukemia with CMR and ASRs of 2.0 and 1.6 per 100 000 people in 2013 and 4.0 and 3.4 per 100 000 people in 2021 (respectively: *P* = 0.002 and *P* < 0.001). However, HL was the only hematological neoplasm with consistent rates over the nine years, which had CMR and ASRs of 0.8 per 100 000 people in 2013 and 0.9 and 0.7 per 100 000 people in 2021, respectively (*P* = 0.247) (Table 2).

**Table 2:** Mortality Trends for Each Hematological Neoplasm by Study Years in Babol (Iran) From 2013-2021

**Years**	**Rate**	**Hodgkin Lymphoma**	**Non-Hodgkin Lymphoma**	**Multiple Myeloma**	**Leukemia**
2013	Crude	0.8	0.2	0.2	2.0
	Standardized (95% CI)	0.8 (0 – 1.5)	0.2 (0 – 0.5)	0.2 (0 – 0.6)	1.6 (0.6 – 2.6)
2014	Crude	1.7	1.2	0.2	0.4
	Standardized (95% CI)	1.5 (0.5 – 2.6)	1.0 (0.2 – 1.9)	0.2 (0 – 0.5)	0.4 (0 – 0.9)
2015	Crude	0.6	3.8	0.2	1.1
	Standardized ( 95% CI )	0.6 (0 – 1.4)	3.3 (1.8 – 4.8)	0.1 (0 – 0.3)	1.0 (0.2 – 1.9)
2016	Crude	0.9	2.8	0.8	3.6
	Standardized ( 95% CI )	0.6 (0.6 – 2.6)	2.4 (1.1 – 3.6)	0.6 (0 – 1.1)	3.1 (1.7 – 4.6)
2017	Crude	0.2	5.2	1.5	1.9
	Standardized ( 95% CI )	0.2 (0 – 0.5)	4.6 (2.9 – 6.4)	1.4 (0.4 – 2.4)	1.6 (0.6 – 2.6)
2018	Crude	1.1	4.0	1.5	3.1
	Standardized ( 95% CI )	1.0 (0.2 – 1.8)	3.6 (2.0 – 5.1)	1.5 (0.4 – 2.5)	3.0 (1.5 – 4.5)
2019	Crude	1.3	4.0	1.6	4.3
	Standardized ( 95% CI )	1.2 (0.3 – 2.1)	3.5 (2.0 – 5.0)	1.4 (0.5 – 2.3)	3.7 (2.2 – 5.7)
2020	Crude	0.1	2.7	0.9	2.8
	Standardized ( 95% CI )	0.1 (0 – 0.4)	2.2 (1.1 – 3.4)	0.7 (0.1 – 1.4)	2.5 (1.2 – 3.7)
2021	Crude	0.9	1.9	1.2	4.0
	Standardized ( 95% CI )	0.7 (0.1 – 1.3)	1.7 (0.7 – 2.6)	1.1 (0.3 – 2.0)	3.4 (2.0 – 4.8)
P-trend		0.247	0.033	0.002	< 0.001

CI: confidence interval.

## Discussion

 Using the Iranian Ministry of Health data, this study examined cancer mortality trends from 2013 to 2021 in Babol. Overall, 357 deaths (10.8%) of all cancer-related deaths were attributed to hematological neoplasms, with an average age of 61.9 ± 19.3 years. The crude and age-adjusted mortality rates of hematological neoplasms showed an increasing trend from 3.1 and 2.7 per 100 000 population in 2013 to 8.1 and 6.9 per 100 000 population in 2021, respectively. Also, both men and women experienced significant accelerations in the trend with each decade of their lives. Furthermore, the analysis of the trend for each hematological neoplasm over the 9-year period revealed an increasing trend for all neoplasms except for HL, which showed a consistent trend. NHL was the most common hematological neoplasm, accounting for 140 (39.2%) deaths.

 The results of our study differ from global trends reported in previous studies. According to a recent analysis, the ASMR for all hematologic cancers has been declining over the past three decades. As of 1990, there were 5.82, 1.4, 3.15, and 0.61 ASMRs per 100 000 population for leukemia, multiple myeloma, NHL, and HL, respectively. This decreased to 4.26 (estimated annual percentage changes (EAPC) = -1.15), 1.42 (EAPC = -0.07), 3.19 (EAPC = -0.09), and 0.34 (EAPC = -2.08) in 2019.^20^

 As a major cause of cancer deaths worldwide, NHL is the eleventh most commonly diagnosed malignancy.^2^ According to the latest results published by the National Cancer Registry in Iran, its age-standardized incidence rate is 0.49 and 0.37 for men and women, respectively.^21^ The increasing trend observed in NHL mortality in our study does not align with the results of other studies. According to Chu et al, the mortality trend for this group of lymphomas has decreased in most regions, except for a few developing countries like Ecuador. Although the observed trend in our study area differs from the global trend, the ASMR of NHL at the end of our study period is still lower than the mortality rate in neighboring countries in West Asia (3.3 per 100 000) in 2020 and close to the reported mortality rate for middle-income countries (1.8 per 100 000).^22^ A meta-analysis using individual patients data identified risk factors for NHL based on its various subgroups, showing that medical and family history, lifestyle, and occupational factors are associated with one or more NHL subgroups.^23^ However, to our knowledge, no study has identified the prevalence of these risk factors in patients in our study area or their association with mortality in hospitalized patients in our region. Therefore, determining the cause of this worrying increasing trend requires further studies.

 Leukemia is among the top ten leading cancers causing death (3.1% of death causes).^2^ This cancer has two major subgroups based on disease progression: acute leukemia and chronic leukemia, with epidemiology varying by subgroup.^24^ The observed trend in leukemia mortality in our study differs from the global and national trends. According to estimates from one study, the mortality trend for leukemia for both genders from 1980 to 2017 has decreased in most countries while it has increased in four countries, including the Philippines, Ecuador, Belarus, and Thailand.^25^ Another study using the Global Burden of Disease (GBD) data reported that despite a 1.5-fold increase in leukemia deaths from 1990 to 2019 in Iran, the ASMR for these cancers decreased from 8.3 in 1990 to 6 in 2019. The highest ASMR in Iran was observed in Khorasan Razavi, East Azerbaijan, and Fars provinces.^26^ In contrast to these results, a study on leukemia mortality trends in Iran from 1995 to 2004 by Fazeli et al observed an increasing trend.^27^

 The ASMR for multiple myeloma shows different trends among countries with varying Sustainable Development Index (SDI) levels. The mortality rate for high SDI quintile countries peaked around 2000 and has since declined. This trend is also observed in upper-middle SDI countries, with a recent peak in ASMR around 2005 followed by a decline. ASMR is increasing in other SDI quintiles.^28^ The ASMR for multiple myeloma in 2018 was 1.3 and 0.9 per 100 000 population for men and women, respectively, which is close to the rate found in our study. Risk factors for this disease include older age, male gender, race, and a positive family history in first-degree relatives.^29^ A study analyzing the incidence and mortality data for HL showed a general decreasing trend for this malignancy over the past decade, with significant associations found between some risk factors and mortality, including obesity, smoking, and low Human Development Index (HDI) levels.^30^

 Mitigating the modifiable risk factors is crucial for reducing the incidence and mortality of hematological malignancies. Additionally, increasing research efforts into new treatments and strengthening surveillance systems to monitor disease patterns and inform policy decisions will be essential.^31^ Our study emphasizes the need for a multifaceted approach to tackling the growing burden of these cancers in northern Iran, including efforts to improve access to cancer care, enhance surveillance in families with blood malignancies and reduce modifiable risk factors.^32-35^

 Investigating the impact of genetic, environmental, and lifestyle factors on the incidence and mortality of hematological neoplasms in this region can provide insights into preventive and targeted therapeutic strategies. Furthermore, assessing patient access and the quality of cancer care in northern Iran can help identify critical areas for improving healthcare.

 This study had some limitations: First, registration and diagnostic failure bias may underestimate neoplasm mortality. This includes patients who died without a diagnosis in the advanced stages of the disease.^36^ Second, the available variables were limited. This restricted data did not allow us to assess the influence of essential factors on mortality, such as tumour histology or stage, treatment types, and socioeconomic status. Consequently, we only analyzed the data based on gender and age. We suggest that cancer registries collect data on cancer characteristics, patient demographics and therapeutic information, where applicable, as this could enhance our understanding of trends in future studies and aid in designing targeted interventions. Third, the study’s geographical and population focus on northern Iran limits the generalizability of the findings. Fourth, the increase in reported cases might be due to improvements in diagnostic techniques over time; therefore, caution is necessary when interpreting the results.

## Conclusion

 Our study concludes that from 2013 to 2021, the mortality trend of hematological malignancies in Babol city has been increasing, regardless of age or gender. It also emphasizes the need for effective cancer prevention and control strategies, including efforts to improve access to cancer care, enhance surveillance in families with blood malignancies and reduce modifiable risk factors. Further research is necessary to identify high-risk populations, understand risk factors, and develop targeted interventions. According to this study, cancer continues to grow in northern Iran and poses a challenge to public health. Furthermore, we emphasize the need for comprehensive national cancer control programs that include prevention, treatment, and palliative care.
